# GHK and DNA: Resetting the Human Genome to Health

**DOI:** 10.1155/2014/151479

**Published:** 2014-09-11

**Authors:** Loren Pickart, Jessica Michelle Vasquez-Soltero, Anna Margolina

**Affiliations:** Skin Biology, Research & Development Department, 4122 Factoria Boulevard SE, Suite No. 200, Bellevue, WA 98006, USA

## Abstract

During human aging there is an increase in the activity of inflammatory, cancer promoting, and tissue destructive genes plus a decrease in the activity of regenerative and reparative genes. The human blood tripeptide GHK possesses many positive effects but declines with age. It improves wound healing and tissue regeneration (skin, hair follicles, stomach and intestinal linings, and boney tissue), increases collagen and glycosaminoglycans, stimulates synthesis of decorin, increases angiogenesis, and nerve outgrowth, possesses antioxidant and anti-inflammatory effects, and increases cellular stemness and the secretion of trophic factors by mesenchymal stem cells. Recently, GHK has been found to reset genes of diseased cells from patients with cancer or COPD to a more healthy state. Cancer cells reset their programmed cell death system while COPD patients' cells shut down tissue destructive genes and stimulate repair and remodeling activities. In this paper, we discuss GHK's effect on genes that suppress fibrinogen synthesis, the insulin/insulin-like system, and cancer growth plus activation of genes that increase the ubiquitin-proteasome system, DNA repair, antioxidant systems, and healing by the TGF beta superfamily. A variety of methods and dosages to effectively use GHK to reset genes to a healthier state are also discussed.

## 1. Introduction

According to the Administration on Aging (http://www.aoa.gov/), there were 39 million people aged 65 and older in 2009 which constituted 12% of the American population. By 2030 it is expected that 19% of the population will be over 65. With life expectancy continuing to increase, we may expect that this trend is here to stay. Unfortunately, with advanced age comes not only wisdom but also many age-related pathological conditions that account for the high rates of hospitalization, increased cost of health care and decreased quality of life. Today, more than ever, there is an urgent need to find safe, easy-to-administer, cost-effective methods, which could not only delay the onset of the age related diseases, but also restore health.

It now becomes increasingly clear that the primary cause of human aging and its attendant diseases is changes in the activity of the human genome. During aging there is an increase in the activity of inflammatory, cancer promoting, and tissue destructive genes plus a decrease in the activity of regenerative and reparative genes [1].

The most exciting discovery of the past decades is that these changes in gene activity can be reversed, often by quite simple and natural molecules [2]. Recent discoveries on the actions of the human tripeptide GHK (glycyl-L-histidyl-L-lysine) to reset gene expression of human cells to a more healthy state may open a door to the therapeutic resetting of genes in the elderly. This can be useful as a preventative measure and a complimentary treatment for conditions typically associated with aging such as cancer, Alzheimer's, chronic obstructive lung disease (COPD), nephropathy, and retinopathy.

GHK was discovered during studies comparing the effect of human plasma from young persons (age 20–25) to plasma from older persons (age 50–70) on the functioning of incubated slices of human hepatic tissue. The younger plasma more effectively induced a profile associated with youth by suppressing fibrinogen synthesis. The active factor was found to be GHK. Since then numerous studies over the course of four decades demonstrated that this simple molecule improves wound healing and tissue regeneration (skin, hair follicles, bones, stomach, intestinal linings, and liver), increases collagen and glycosaminoglycans, stimulates synthesis of decorin, increases angiogenesis, and nerve outgrowth; possesses antioxidant and anti-inflammatory effects, and increases cellular stemness and the secretion of trophic factors by mesenchymal stem cells [3–6].

GHK's actions on gene expression were determined by the Broad Institute and, using their data, we determined that GHK increased or decreased gene expression (UP or DOWN more than 50%) in 32.1% of the human genes [7]. In a recent gene study, the Broad Institute's Connectivity Map was used to find potential therapeutic agents for aggressive, metastatic colon cancer [8]. The gene analysis computer program selected GHK from 1,309 bioactive molecules as the best choice to reset the diseased gene patterns to a healthier pattern. When three lines of human cancer cells (SH-SY5Y neuroblastoma cells, U937 histolytic cells, breast cancer cells) were incubated in culture with 1 to 10 nanomolar GHK, the programmed cell death system (apoptosis) was reactivated and cell growth inhibited [9]. When cells derived from the damaged areas of the lungs of COPD patients were incubated with 10 nanomolar GHK, the tripeptide recapitulated TGF beta induced genes expression patterns which led to the organization of the actin cytoskeleton and elevated the expression of integrin. This restored proper collagen contraction and remodeling by lung fibroblasts [10]. These results, combined with GHK's broad spectrum of positive actions on many systems that maintain human health, suggest that therapies using GHK might provide health benefits to the elderly.

In this paper, we discuss the following actions of GHK on genes important in healthy aging.

*(1) The Suppression of Fibrinogen Synthesis.* Fibrinogen is an excellent predictor of mortality especially in patients with cardiovascular complications [11, 12]. GHK was isolated as a plasma factor that suppressed fibrinogen synthesis in liver tissue and in mice.

*(2) Activation of the Ubiquitin/Proteasome System (UPS).* The UPS removes damaged proteins. Higher activities of the UPS appear to retard aging effects [13, 14].

*(3) Activation of DNA Repair Genes*. DNA damage is promptly repaired in young and healthy cells, however, as we age, DNA damage starts accumulating. Resetting activity of DNA repair genes can diminish deleterious effects of aging.

*(4) Antioxidant Genes.* Free radicals and toxic end products of lipid peroxidation are linked to atherosclerosis, cancer, cataracts, diabetes, nephropathy, Alzheimer's disease and other severe pathological conditions of aging.

*(5) Suppression of Insulin and Insulin-Like Genes.* The insulin family has been proposed as a negative controller of longevity; higher levels of insulin and insulin-like proteins reduce the lifespan [15].

*(6) Tissue Repair by TGF Superfamily.* General tissue repair by the TGF superfamily as exemplified by COPD (chronic obstructive pulmonary disease).

*(7) Cancer Controlling Genes*. Caspase, growth regulatory, and DNA repair genes are important in cancer suppression.

In addition to discussing GHK actions in this paper, we suggest administrative methods and dosages that should be effective in humans.

## 2. Methods and Results

### 2.1. Gene Expression Analysis

The connectivity map was used to acquire our gene expression data (retrieved March 5, 2013) [16]. Within the Connectivity Map repository there are three GHK gene signatures. Each signature was produced using the GeneChip HT Human Genome U133A Array. GHK was tested on 2 of the 5 cell lines used by the Connectivity Map. Two of the profiles were created using the PC3 cell line while the third used the MCF7 cell line. Our studies utilized all three gene expression profiles.

This genomic data was then analyzed using GenePattern [17]. The CEL files were processed with MAS5 and background correction. Files were then uploaded to the ComparativeMarkerSelectionViewer module in order to view fold changes for each probe set.

Since many probe sets map to the same gene we converted the fold changes in m-RNA production produced by GenePattern to percentages and then averaged all probe sets representing the same gene. It was determined that the 22,277 probe sets in the Broad data represent 13,424 genes. This ratio (1.66) was used to calculate the overall number of genes that are affected by GHK at various cutoff points (rather than probe sets). The number of genes stimulated or suppressed by GHK with a change greater than or equal to 50% is 31.2%.

### 2.2. Fibrinogen Suppression

Fibrinogen consists of three polypeptide chains; alpha, beta, and gamma. GHK strongly suppresses the gene for the beta chain of fibrinogen. A lack of adequate FGB will effectively stop fibrinogen syntheses since equal amounts of all three polypeptide chains are needed to produce fibrinogen. See Table 1.

GHK also suppresses the production of the inflammatory cytokine interleukin-6 (IL-6) which is a main positive regulator of fibrinogen synthesis, through its interaction with fibrinogen genes [18]. In cell culture systems, GHK suppresses IL-6 secretion in skin fibroblasts and IL-6 gene expression in SZ95 sebocytes [19, 20].

In summary, the effects of GHK on the FGB gene plus its effects on IL-6 production imply a suppression of overall fibrinogen production.

### 2.3. Ubiquitin/Proteasome System

GHK stimulated gene expression in 41 UPS genes while suppressing only 1 UPS gene. See Table 2.

### 2.4. DNA Repair Genes

GHK was primarily stimulatory for DNA repair genes (47 UP, 5 DOWN). See Tables 3 and 4.

### 2.5. Antioxidant Genes

Among the 13,424 available genes in the Broad Institute data, we were able to identify 14 antioxidant genes in which GHK stimulates as well as two prooxidant genes that GHK suppresses. GHK increases the expression of the oxidative/inflammatory gene NF-*κ*B2 103% but also increases the expression of two inhibitors of NF-*κ*B, TLE1 by 762% and IL18BP by 295%, thus possibly inhibiting the activity of the NF-*κ*B protein. See Table 5.

### 2.6. Insulin and Insulin-Like System

GHK stimulates 3 genes in this system and suppresses 6 genes. See Table 6.

## 3. Discussion

Even though numerous and diverse beneficial effects of GHK have been known for decades, it was not clear how one simple molecule could accomplish so much. The use of gene expression data greatly extends our understanding of GHK's effects and its potential treatments of some of the diseases and biochemical changes associated with aging. As a potential therapeutic agent GHK has a clear advantage over many other active chemicals that may also show promising results in gene profiling experiments, its gene modulating effects correspond to findings from* in vivo* experiments. When GHK is administered internally to an animal, it induces actions throughout the body.

The treatment of rats, mice, and pigs with GHK was shown to effectively activate systemic healing throughout the animal. For example, if GHK is injected into the thigh muscles of rats, it induces accelerated healing in implanted Hunt-Schilling wound chambers. If the GHK is injected into the thigh muscles of mice, it accelerates the healing of an experimental full thickness surgical defect wound model on its back. If injected into thigh muscles of pigs, it induces accelerates healing of full thickness surgical defect wounds on its back [37]. If GHK is injected intraperitoneally into rats, it heals tubular bone fractures [38]. Wound healing requires activation of gene expression for numerous pathways and wound healing data confirms that GHK is able to activate gene expression in animals [39–45].

There is still not enough information to translate gene profiling data into biological effects. However, based on the documented activity of GHK* in vivo*, we can predict the following beneficial actions from our gene profiling data.

### 3.1. Fibrinogen

Fibrinogen, the protein which is used to make blood clots, is also a strong predictor of mortality in cardiovascular patients. After vascular incidents, such as myocardial infarction, fibrinogen concentrations increase sharply. The free, unclotted fibrinogen protein increases the “stickiness” of red blood cells which stack together forming rouleaux. This increases the time of the “solid” blood state which decreases blood flow through the microcirculation where blood flows like a thixotropic fluid, switching between a solid phase and a liquid phase, somewhat like toothpaste. As a solid, it stops oxygen and nutrient flow to the tissues. This, in itself, can cause tissue damage.

The gene data on GHK's suppression of FGB (the fibrinogen beta chain) combined with its actions on lowering IL-6 secretion on fibroblasts and sebocytes appears to be sufficient to explain its lowering effect on fibrinogen.

### 3.2. Ubiquitin Proteasome System

The ubiquitin proteasome system (UPS) functions in the removal of damaged or misfolded proteins. Aging is a natural process that is characterized by a progressive accumulation of unfolded, misfolded, or aggregated proteins. In particular, the proteasome is responsible for the removal of normal as well as damaged or misfolded proteins. Recent work has demonstrated that proteasome activation by either genetic means or use of compounds retards aging [13, 14].

In our screening of UPS genes with a percent change of at least ±50%, GHK increased gene expression in 41 UPS genes while suppressing 1 UPS gene. Thus, it should have a positive effect on this system [13, 14, 46].

### 3.3. DNA Repair

It is estimated that normal metabolic activities and environmental factors such as UV light and radiation can cause DNA damage, resulting in somewhere between 1000 and as many as 1 million individual molecular lesions per cell per day. Lack of sufficient DNA repair is considered a cause of cell senescence, programmed cell death, and unregulated cell division, which can lead to the formation of a tumor that is cancerous [47–50].

GHK was stimulatory for DNA repair genes (47 stimulated, 5 suppressed) suggesting an increased DNA repair activity.

### 3.4. Antioxidant Defense

Free radicals and toxic end products of lipid peroxidation are linked to atherosclerosis, cancer, cataracts, diabetes, nephropathy, Alzheimer's disease, and other severe pathological conditions of aging. Reactive oxygen species (ROS) and reactive carbonyl species (RCS) are produced in cells in small quantities under physiological conditions and play an important role in cell signaling and immune defense. A robust antioxidant network maintains balance between free radical production and scavenging, ensuring that the overall damage from free radicals is low. However, in the course of aging and in pathological conditions such as inflammation, the balance may shift toward free radical accumulation that can lead to oxidative stress and eventually to cell death [51].

GHK increases gene expression of 14 antioxidant genes and suppresses the expression of 2 prooxidant genes. It increases the expression of the oxidative/inflammatory gene NF-*κ*B2 103% but also increases the expression of two inhibitors of NF-*κ*B, TLE1 by 762% and IL18BP by 295%; thus, it possibly inhibits the activity of the NF-*κ*B protein.

GHK also possesses antioxidant activities in cell culture and* in vivo*.

In dermal wound healing in rats, GHK, attached to biotin to bind it to collagen pads covering wounds, produced a higher production of protein antioxidants in the wound tissue. Superoxide dismutase was increased 80% while catalase was increased 56% [52, 53]. GHK reduced gastric mucosal damage by 75% against lipid peroxidation by oxygen-derived free radicals induced by acute intragastric administration of ethanol [54].

Interleukin 1 beta can induce serious oxidative damage to cultured cells [55, 56]. GHK markedly reduced oxidative damage by interleukin 1-beta to cultured insulin secreting pancreatic cells [57].

In another study, GHK entirely blocked the extent of* in vitro* Cu(2+)-dependent oxidation of low density lipoproteins (LDL). Treatment of LDL with 5 microM Cu(2+) for 18 hours in phosphate buffered saline (PBS) resulted in extensive oxidation as determined by the content of thiobarbituric acid reactive substances. Oxidation was entirely blocked by GHK. In comparison, copper, zinc-superoxide dismutase provided only 20% protection [58].

Acrolein, a well-known carbonyl toxin, is produced by lipid peroxidation of polyunsaturated fatty acids. GHK directly blocks the formation of 4-hydroxynonenal and acrolein toxins created by carbonyl radicals that cause fatty acid decomposition [59, 60]. GHK also blocks lethal ultraviolet radiation damage to cultured skin keratinocytes by binding and inactivating reactive carbonyl species such as 4-hydroxynoneal, acrolein, malondialdehye, and glyoxal [61].

Iron has a direct role in the initiation of lipid peroxidation. An Fe(2+)/Fe(3+) complex can serve as an initiator of lipid oxidation. The major storage site for iron in serum and tissue is ferritin and the superoxide anion can promote the mobilization of iron from ferritin which can catalyze lipid peroxidation. GHK : Cu(2+) produced an 87% inhibition of iron release from ferritin by apparently blocking iron's exit channels from the protein [62].

### 3.5. Insulin and Insulin-Like Pathways

The insulin/IGF-1-like receptor pathway is a contributor to the biological aging process in many organisms. The gene expression data suggests that GHK suppresses this system as 6 of 9 of the affected insulin/IGF-1 genes are suppressed.

Insulin/IGF-1-like signaling is conserved from worms to humans.* In vitro* experiments show that mutations that reduce insulin/IGF-1 signaling have been shown to decelerate the degenerative aging process and extend lifespan in many organisms, including mice and possibly humans. Reduced IGF-1 signaling is also thought to contribute to the “antiaging” effects of calorie restriction [63].

### 3.6. COPD

COPD (chronic obstructive lung disease) is a leading cause of death in the world. It is a deadly and painful disease of the lungs that causes difficulty in breathing. In people with COPD, the tissues necessary to support the physical shape and function of the lungs are destroyed. COPD is most often caused by tobacco smoking and long-term exposure to air pollution but is also a component of normal aging. As the lungs get older, the elastic properties decrease, and the tensions that develop can result in areas of emphysema.

The most explored of GHK's actions is the repair of damaged tissues (skin, hair follicles, stomach and intestinal linings, and boney tissue) either by the use of copper-peptide containing creams or by induction of systemic healing. Campbell et al. found that GHK's resetting of gene expression of fibroblasts from COPD patients fits into this category of tissue repair via the TGF beta superfamily. Campbell et al. found that GHK directly increases TGF beta and other family members which activate the repair process [10].

Treatment of human fibroblasts with GHK recapitulated TGF beta-induced gene expression patterns, led to the organization of the actin cytoskeleton and elevated the expression of integrin beta1. Furthermore, addition of GHK or TGF beta restored collagen I contraction and remodeling by fibroblasts derived from COPD lungs compared to fibroblasts from former smokers without COPD.

On another note, persons with severe COPD use air inhalation systems that pump misty, water-filled air in and out of the lungs. Often steroids are added to the solution to suppress the lung inflammation, while this provides short-term help, it also inhibits lung repair. In theory, GHK could be infused into the blood stream of patients to repair the lung tissue, added to a misting solution or used in combination of a carrier like DMSO along with GHK (use a 1 : 1 molar ratio of GHK to DMSO). DMSO and GHK or GHK : Cu(2+) has always worked well together on wound healing. DMSO has been used in the past as a treatment for COPD, so there should be few safety issues.

Also, it may be possible to induce more extensive rebuilding of lung tissue. The mixture of GHK, transferrin, and somatostatin was sufficient to promote branching in the absence of serum in organ culture, all of which could be added to the misting solution [64].

### 3.7. Cancer

In 2010, Hong et al. identified 54 genes associated with aggressive, metastatic, human colon cancer [8]. The Broad Institute's Connectivity Map was used to find compounds that reverse the differential expressions of these genes. The results indicated that two wound healing and skin remodeling molecules, GHK at 1 micromolar and securinine at 18 micromolar, could significantly reverse the differential expression of these genes and suggested that they may have a therapeutic effect on the metastasis-prone patients.

Normal healthy cells have checkpoint systems to self-destruct if they are synthesizing DNA incorrectly through programmed cell death or the apoptosis system. Matalka et al. demonstrated that GHK, at 1 to 10 nanomolar, reactivated the apoptosis system, as measured by caspases 3 and 7, and inhibited the growth of human SH-SY5Y neuroblastoma cells, human U937 histiocytic lymphoma cells, and human breast cancer cells [9]. In contrast, the GHK accelerated the growth of healthy human NIH-3T3 fibroblasts.

Our analysis of GHK's actions found that it increased gene expression in 6 of the 12 human caspase genes that activate apoptosis. In 31 other genes, GHK altered the pattern of gene expression in a manner that would be expected to inhibit cancer growth. In DNA repair genes there was an increase (47 UP, 5 DOWN) [7]. These results support the idea that GHK may help slow or suppress cancer growth.

Linus Pauling's group once used a copper tripeptide, Gly-Gly-His : Cu(2+) and ascorbic acid as a cancer treatment method. In a recent paper, we used their basic method but with GHK : Cu(2+) and ascorbic acid, which strongly suppressed sarcoma 180 in mice without any evident distress to the animals [7]. GHK altered gene expression in 84 genes (caspases, cytokines, and DNA repair genes) in a manner that would be expected to suppress cell growth. On skin, GHK seems to act most strongly in the late stage of healing, called remodeling, where cellular migration into the wound area is stopped and cellular debris is removed. The anticancer actions of small copper peptides may be a side effect of this system.

The use of GHK : Cu(2+) and ascorbic acid should be investigated in more detail. The mice treated in this manner appeared to remain very healthy and active, in contrast to the toxicities of current cancer chemotherapy.

### 3.8. GHK as a Clinical Treatment

GHK, abundantly available at low cost in bulk quantities, is a potential treatment for a variety of disease conditions associated with aging. The molecule is very safe and no issues have ever arisen during its use as a skin cosmetic or in human wound healing studies.

GHK has a very high affinity for Cu(2+) (pK of association = 16.4) and can easily obtain copper from the blood's albumin bound Cu(2+) (pK of association = 16.2) [3]. Most of our key experiments used a 1 : 1 mixture of copper-free GHK and GHK : Cu(2+). In wound healing experiments, the addition of copper strongly enhanced healing. However, others often obtain effective results without added copper.

Cells within tissues are under the influence of many other regulatory molecules. Thus, GHK would be expected to influence the cells' gene expression to be more similar to that of a person of age 20–25, an age when the afflictions of aging are very rare. Based on our studies, in which GHK was injected intraperitoneally once daily to induce systemic wound healing throughout the body, we estimate about 100–200 mgs of GHK will produce therapeutic actions in humans. But even this may overestimate the necessary effective dosage of the molecule. Most cultured cells respond maximally to GHK at 1 nanoM. GHK has a half-life of about 0.5 to 1 hour in plasma and two subsequent tissue repair studies in rats found that injecting GHK intraperitoneally 10 times daily lowered the necessary dosage by approximately 100-fold in contrast to our earlier studies [38, 65].

The most likely effective dosage of GHK was given to rats for healing bone fractures. This mixture of small molecules included Gly-His-Lys (0.5 *μ*g/kg), dalargin (1.2 *μ*g/kg) (an opioid-like synthetic drug), and the biological peptide thymogen (0.5 *μ*g/kg) (L-glutamyl-L-tryptophan) to heal bones. The total peptide dosage is about 2.2 *μ*g/kg or, if scaled for the human body, about 140 *μ*g per injection with 10 treatments per day [38, 65].

The use of portable continuous infusion pumps for a treatment might maintain an effective level in the plasma and extracellular fluid with the need for much less GHK. Possibly the peptide could be administered with a transdermal patch [66]. Another approach could be to use peptide-loaded liposomes as an oral delivery system for uptake into the intestinal wall without significant breakdown [67, 68].

## 4. Conclusion

Most current theories and therapies to treat disease tend to target only one biochemical reaction or pathway. But for human aging, our data suggests that we must think of simultaneously resetting hundreds to thousands of genes to protect at-risk tissues and organs. GHK may be a step towards this gene resetting goal.

## Figures and Tables

**Table 1:** GHK and fibrinogen.

Gene title	Percent change in gene expression
Fibrinogen alpha chain, FGA	121
Fibrinogen beta chain, FGB	−475

**Table 2:** Ubiquitin/proteasome system and GHK.

Up	Gene title	Percent change in gene expression
1	Ubiquitin specific peptidase 29, USP29	1056
2	Ubiquitin protein ligase E3 component n-recognin 2, UBR2	455
3	Gamma-aminobutyric acid (GABA) B receptor, 1 /// ubiquitin D, GABBR1 /// UBD	310
4	Ubiquitin specific peptidase 34, USP34	195
5	Parkinson protein 2, E3 ubiquitin protein ligase (parkin), PARK2	169
6	Ubiquitin-conjugating enzyme E2I (UBC9 homolog, yeast), UBE2I	150
7	Ubiquitin protein ligase E3 component n-recognin 4, UBR4	146
8	Ubiquitin protein ligase E3B, UBE3B	116
9	Ubiquitin specific peptidase 2, USP2	104
10	Ubiquitin-like modifier activating enzyme 6, UBA6	104
11	Ubiquitination factor E4B (UFD2 homolog, yeast), UBE4B	97
12	Ubiquitin-conjugating enzyme E2M (UBC12 homolog, yeast), UBE2M	92
13	Ubiquitin-like modifier activating enzyme 7, UBA7	88
14	HECT, C2 and WW domain containing E3 ubiquitin protein ligase 1, HECW1	81
15	Proteasome (prosome, macropain) 26S subunit, ATPase, 3, PSMC3	81
16	Ubiquitin-conjugating enzyme E2D 1 (UBC4/5 homolog, yeast), UBE2D1	79
17	Proteasome (prosome, macropain) subunit, beta type, 2, PSMB2	79
18	Ubiquitin protein ligase E3 component n-recognin 5, UBR5	77
19	Ubiquitin specific peptidase 21, USP21	76
20	OTU domain, ubiquitin aldehyde binding 2, OTUB2	76
21	Proteasome (prosome, macropain) inhibitor subunit 1 (PI31), PSMF1	75
22	Ubiquitin-conjugating enzyme E2H (UBC8 homolog, yeast), UBE2H	73
23	Ubiquitin-conjugating enzyme E2N (UBC13 homolog, yeast), UBE2N	72
24	Ubiquitin carboxyl-terminal hydrolase L5, UCHL5	71
25	Proteasome (prosome, macropain) 26S subunit, non-ATPase, 13, PSMD13	70
26	Ubiquitin associated protein 1, UBAP1	70
27	Ubiquitin-conjugating enzyme E2B (RAD6 homolog), UBE2B	69
28	TMEM189-UBE2V1 readthrough /// ubiquitin-conjugating enzyme E2 variant 1, TMEM189-UBE2V1 /// UBE2V1	67
29	Proteasome (prosome, macropain) 26S subunit, non-ATPase, 1, PSMD1	64
30	Proteasome (prosome, macropain) 26S subunit, non-ATPase, 3, PSMD3	64
31	Ariadne homolog, ubiquitin-conjugating enzyme E2 binding protein, 1 (drosophila), ARIH1	61
32	BRCA1 associated protein-1 (ubiquitin carboxy-terminal hydrolase), BAP1	60
33	Ubiquitin interaction motif containing 1, UIMC1	60
34	Ubiquitin associated protein 2-like, UBAP2L	57
35	Ubiquitin protein ligase E3 component n-recognin 7 (putative), UBR7	56
36	Ubiquitin-conjugating enzyme E2G 1 (UBC7 homolog, yeast), UBE2G1	54
37	Itchy E3 ubiquitin protein ligase homolog (mouse), ITCH	54
38	Ubiquitin-conjugating enzyme E2D 4 (putative), UBE2D4	51
39	Proteasome (prosome, macropain) 26S subunit, non-ATPase, 10, PSMD10	50
40	WW domain containing E3 ubiquitin protein ligase 1, WWP1	50
41	Ubiquitin-like 3, UBL3	50

Down	Gene title	Percent change in gene expression

1	Ubiquitin associated and SH3 domain containing A, UBASH3A	−89

**Table 3:** GHK and DNA repair.

Percent change in gene expression	Genes up	Genes down
50%–100%	41	4
100%–150%	2	1
150%–200%	1	0
200%–250%	2	0
250%–300%	1	0

**Table 4:** The most affected DNA repair genes.

Up	Gene title	Percent change in gene expression
1	Poly (ADP-ribose) polymerase family, member 3, PARP3	253
2	Polymerase (DNA directed), mu, POLM	225
3	MRE11 meiotic recombination 11 homolog A MRE11A	212
4	RAD50 homolog (S. cerevisiae), RAD50	175
5	Eyes absent homolog 3 (Drosophila), EYA3	128
6	Retinoic acid receptor, alpha, RARA	123

Down	Gene title	Percent change in gene expression

1	Cholinergic receptor, nicotinic, alpha 4, CHRNA4	−105

**Table 5:** GHK effects on antioxidant genes.

Up	Genes	Percent change in gene expression	Comments
1	TLE1	762	Inhibits the oxidative/inflammatory gene NF-*κ*B [21].
2	SPRR2C	721	This proline-rich, antioxidant protein protects outer skin cells from oxidative damage from ROS. When the ROS level is low, the protein remains in the outer cell membrane but when the ROS level is high, the protein clusters around the cell's DNA to protect it [22, 23].
3	ITGB4	609	Upregulation of ITGB4 promotes wound repair ability and antioxidative ability [24].
4	APOM	403	Binds oxidized phospholipids and increases the antioxidant effect of HDL [25].
5	PON3	319	Absence of PON3 (paraoxonase 3) in mice resulted in increased rates of early fetal and neonatal death. Knockdown of PON3 in human cells reduced cell proliferation and total antioxidant capacity [26].
6	IL18BP	295	The protein encoded by this gene is an inhibitor of the proinflammatory cytokine IL18. IL18BP abolished IL18 induction of interferon-gamma (IFNgamma), IL8, and activation of NF-*κ*B *in vitro*. Blocks neutrophil oxidase activity [27].
7	HEPH	217	Inhibits the conversion of Fe(2+) to Fe(3+). HEPH increases iron efflux, lowers cellular iron levels, suppresses reactive oxygen species production, and restores mitochondrial transmembrane potential [28].
8	FABP1	186	Reduces intracellular ROS level. Plays a significant role in reduction of oxidative stress [29, 30].
9	PON1	149	PON1 (paraoxonase 1) is a potent antioxidant and a major anti-atherosclerotic component of high-density lipoprotein [31].
10	MT3	142	Metallothioneins (MTs) display *in vitro *oxyradical scavenging capacity, suggesting that they may specifically neutralize hydroxyl radicals. Metallothioneins and metallothionein-like proteins isolated from mouse brain act as neuroprotective agents by scavenging superoxide radicals [32, 33].
11	PTGS2	120	Produces cyclooxygenase-II (COX-II) which has antioxidant activities [34].
12	NF-*κ*B2	103	NF-*κ*B, an oxidative/inflammatory protein, is involved in cellular responses to stimuli such as stress, cytokines, free radicals, ultraviolet irradiation, oxidized LDL, and bacterial or viral antigens [21].
13	NFE2L2	56	Nuclear respiratory factor 2 helps activate antioxidant responsive element regulated genes which contribute to the regulation of the cellular antioxidant defense systems [35].
14	PTGS1	50	Produces cyclooxygenase-I (COX-I) which has antioxidant activity [34].

Down	Genes	Percent change in gene expression	Comments

1	IL17A	−1018	This strongly suppressed cytokine can stimulate the expression of IL6 and cyclooxygenase-2 (PTGS2/COX-2), as well as enhancing the production of nitric oxide (NO). High levels of this cytokine are associated with several chronic inflammatory diseases including rheumatoid arthritis, psoriasis, and multiple sclerosis (NCBI GENE entry).
2	TNF	−115	GHK suppresses this prooxidant TNF gene which inhibits the antioxidant IL18 [36].

**Table 6:** GHK and insulin/insulin-like genes.

Up	Gene title	Percent change in gene expression
1	Insulin-like 6, INSL6	188
2	Insulin-like growth factor 2 mRNA binding protein 3, IGF2BP3	136
3	Insulin-like growth factor binding protein 3, IGFBP3	82

Down	Gene title	Percent change in gene expression

1	Insulin-like growth factor 1 (somatomedin C), IGF1	−522
2	Insulin receptor-related receptor, INSRR	−437
3	Insulin, INS	−289
4	Insulin-like 3 (Leydig cell), INSL3	−188
5	Insulin-like growth factor binding protein 7, IGFBP7	−110
6	Insulin-like 5, INSL5	−101
